# Supplementary material for: SyNDI: synchronous network data integration framework
Source: BMC Bioinformatics. 2018 Nov 6;19:403. doi: 10.1186/s12859-018-2426-5 (PMC6219086; doi:10.1186/s12859-018-2426-5)
Supplement: Supplementary file 5 — Files of the example presented in Additional file 4. This additional file is a zip package that contains all files used in Additional file 4. Detailed descriptions of each of these files are presented in a README file included in this zip package. (GZ 30411 kb) [file 12859_2018_2426_MOESM5_ESM.gz › SyNDITools/Meme2Fimo/meme_4.9.0_4.tar.gz!/meme_4.9.0/doc/glam2scan_man.html]

glam2scan Manual


# glam2scan Manual

glam2scan finds matches, in a sequence database, to a motif
discovered by glam2. Each match receives a score, indicating how well it
fits the motif.

## Basic usage

Running glam2scan without any arguments gives a usage message:

```
Usage: glam2scan [options] alphabet my_motif.glam2 my_seqs.fa
Main alphabets: p = proteins, n = nucleotides
Main options (default settings):
-h: show all options and their default settings
-o: output file (stdout)
-n: number of alignments to report (25)
-2: examine both strands - forward and reverse complement
```

glam2scan needs three pieces of information - the alphabet, a
file containing a motif found by glam2, and a file of sequences in FASTA
format:

```
glam2scan p prot_motif.glam2 lotsa_prots.fa
```

```
glam2scan n nuc_motif.glam2 lotsa_nucs.fa
```

An alphabet other than p or n is interpreted as the name of an alphabet file. Motif files from glam2
often contain multiple motifs: glam2scan only considers the top one.

## Output format

The output begins with some general information:

```
GLAM2scan
Version 9999

glam2scan p prot_motif.glam2 lotsa_prots.fa
```

This is followed by motif matches, sorted in order of score. A
motif match looks like this:

```
                 **.****
SOS1_HUMAN   780 HPIE.IA 785 + 8.70
```

The name of the sequence with the match appears on the left; the
start and end coordinates of the match appear on either side of the
matching sequence; the match score appears on the right. The plus sign
indicates the strand of the match (only meaningful when considering both
strands of nucleotide sequences with the -2 option). The stars indicate
the *key positions* of the motif: the alignment of the match to
the key positions is shown.

## Basic options

- Use -o to write the output to a file rather than to the
  screen:

  ```
  glam2scan -o my_matches p prot_motif.glam2 lotsa_prots.fa
  ```
- Use -n to specify how many matches to report:

  ```
  glam2scan -n 100 n nuc_motif.glam2 lotsa_nucs.fa
  ```

  Matches are sorted in order of score. If scores are tied, they are
  sorted in alphabetical order of sequence name. If sequence names are
  also identical, the order is arbitrary.
- Use -2 to search both strands of nucleotide sequences:

  ```
  glam2scan -2 nuc_motif.glam2 lotsa_nucs.fa
  ```

## Advanced options

The remaining options are somewhat specialized. For typical
usage, it is reasonable to set them to exactly the same values as were
used with glam2 to discover the motif.

- Use -D to specify the deletion pseudocount.
- Use -E to specify the 'no-deletion' pseudocount.
- Use -I to specify the insertion pseudocount.
- Use -J to specify the 'no-insertion' pseudocount.
- Use -d to specify a Dirichlet
  mixture file.

## Motif format

Some users may wish to make 'fake' glam2 motifs for input to
glam2scan, for instance based on motifs found by other tools. Most of
the glam2 output is ignored by glam2scan, and a minimal motif file looks
like this:

```
                **..****
seq1         10 HP..D.IG
seq2          5 HPGADLIG
seq3          7 HP..ELIG
seq4          5 HP..ELLA
```

The sequence names and coordinates are ignored, but some
placeholder characters should be present. The stars indicating key
positions are necessary, and the first and last columns must be starred.
